# Ship Detection from Ocean SAR Image Based on Local Contrast Variance Weighted Information Entropy

**DOI:** 10.3390/s18041196

**Published:** 2018-04-13

**Authors:** Weibo Huo, Yulin Huang, Jifang Pei, Qian Zhang, Qin Gu, Jianyu Yang

**Affiliations:** School of Communication and Information Engineering, University of Electronic Science and Technology of China, 2006 Xiyuan Road, Gaoxin Western District, Chengdu 611731, China; yulinhuang@uestc.edu.cn (Y.H.); peijfstudy@126.com (J.P.); cqzhangq92@163.com (Q.Z.); guqin2007@163.com (Q.G.); jyyang@uestc.edu.cn (J.Y.)

**Keywords:** SAR image, ship detection, maximally stable extremal region, local contrast variance weighted information entropy

## Abstract

Ship detection from synthetic aperture radar (SAR) images is one of the crucial issues in maritime surveillance. However, due to the varying ocean waves and the strong echo of the sea surface, it is very difficult to detect ships from heterogeneous and strong clutter backgrounds. In this paper, an innovative ship detection method is proposed to effectively distinguish the vessels from complex backgrounds from a SAR image. First, the input SAR image is pre-screened by the maximally-stable extremal region (MSER) method, which can obtain the ship candidate regions with low computational complexity. Then, the proposed local contrast variance weighted information entropy (LCVWIE) is adopted to evaluate the complexity of those candidate regions and the dissimilarity between the candidate regions with their neighborhoods. Finally, the LCVWIE values of the candidate regions are compared with an adaptive threshold to obtain the final detection result. Experimental results based on measured ocean SAR images have shown that the proposed method can obtain stable detection performance both in strong clutter and heterogeneous backgrounds. Meanwhile, it has a low computational complexity compared with some existing detection methods.

## 1. Introduction

With the development of marine traffic, the number of ships on the high seas has increased greatly. The increased ships can improve seaborne trade. In the report of UNCTAD/RMT/2017, total volumes of seaborne trade reached 10.3 billion tons in 2016, and especially the strong import demand in China continued to support world maritime seaborne trade. However, in recent years, illegal activities such as smuggling, sea-jacking and maritime terrorism have seriously affected maritime trade [1]. Therefore, maritime security is vital to global, regional and national economies. In order to guarantee the safe navigation of the vessel and the safety of sea activities, the surveillance of ocean ships has become a very important issue for coastal countries [2,3]. As a main application of maritime surveillance, ship detection has received more and more attention [4]. At present, the most common and effective ship detection technologies are based on various sensors, such as radar and infrared sensor. In practice, the ship detection system should have the capacity of all-time, all-weather and have a wide-area observation. As synthetic aperture radar (SAR) can provide high-resolution images of the observed ocean day and night and independent of weather condition, ship detection from SAR images is an effective technology and has become a hot research field [5,6].

In general, weak wind results in a smooth sea surface and weak radar backscatter, which appears as a homogeneous background in a SAR image. This situation is advantageous for ship detection. However, due to strong wind generating sea surface ripples, causing a rough sea surface and strong radar backscatter, the sea background in the SAR image is spatially heterogeneous and has high intensity. In this condition, ship targets are often overwhelmed by strong sea clutter, leading to a low signal-to-clutter ratio (SCR). Consequently, ship detection from a SAR image is an extremely tough task.

Many researchers have made efforts to deal with the heterogeneous and strong clutter backgrounds in ship detection [7,8,9,10] and have achieved some valuable results. In addition, the studies of sub-aperture-based [11] and physically-based techniques [12,13,14] in the ship detection field have received much attention recently. Conventional ship detection methods generally utilize a constant false alarm detection (CFAR) technique, such as CA-CFAR, OS-CFAR, etc. [15,16,17]. The CFAR-based methods adopt a sliding window structure and compare the cell under test with a threshold estimated by its surroundings within the window. These methods can obtain considerable detection results under a complex background. However, these methods are seriously dependent on the prior knowledge of the sea background and consume a huge amount of computational resource, leading to the limitation of their application. In order to eliminate the dependence on the prior knowledge, an adaptive detector based on local variance weighed information entropy (VWIE) was proposed [18], which has been proven to be an effective method. The VWIE-based detector can effectively amplify the difference between the target and its surroundings, suppressing sea clutter and the noise [19,20]. However, as the VWIE-based detector adopts the sliding window technique, huge computational consumption is required for large-sized ocean SAR images. Meanwhile, the selection of the sliding window size is also a thorny problem, and the VWIE-based detector is not robust to the SAR images with complex backgrounds. Wang et al. [21] improved the VWIE-based detector using the multiscale local contrast measure (LCM) method [22] to determine the local optimal window size and proposed a multiscale variance weighted information entropy (MVWIE) detection method, which can enhance the robustness of the algorithm. However, the MVWIE method needs a huge number of repeated calculations around a certain pixel region, adding to the extra computational burden sharply. Therefore, the MVWIE method is not suitable for real-time detection from ocean SAR images.

If the detected region can be restricted to a small range, not the whole SAR image, then the detection efficiency can be significantly improved. Based on this analysis, regional pre-screening technology can be employed to quickly achieve potential target regions, which are very smaller compared to the whole SAR image. Then, further exploration can be done in these selected regions to realize accurate ship detection with high efficiency. The maximally-stable extremal region (MSER) method is a region detector used to extract the stable region (called the MSER region) from an image [23]. It turns out that the features of the MSER region are identified with the characteristics of the ship region in a SAR image. Besides, the MSER method has the advantages of stability compared with other region detectors, fast computing and multiscale detection [24], which are very suitable for quick target region extraction in ocean SAR images [25].

Considering the effectiveness and efficiency of ship detection, this paper proposes an innovative ship detection method of SAR images named the MSER-based local contrast variance weighted information entropy (LCVWIE) method. The proposed method can not only achieve a satisfactory detection result in various ocean scenes, but also can reduce the computational time significantly. First, we use the MSER method to extract the ship candidate regions from the original SAR image. Then, the local contrast variance weighted information entropy (LCVWIE) of the extracted candidate region slices is calculated. Finally, the LCVWIE values of the candidate regions are compared with the proposed adaptive threshold to obtain the final detection result.

The proposed MSER-based LCVWIE method does not depend on the a priori knowledge of the background and ship and can achieve quick and accurate ship detection results in various ocean scenes. Experimental results based on different ocean SAR images have shown that the proposed method can obtain a commendable detection performance both in strong clutter and heterogeneous backgrounds; meanwhile, the computational efficiency is significantly improved compared with some existing detection methods.

The remainder of this paper consists of the following sections: Section 2 illustrates the proposed ship detection method, and the experimental results are demonstrated in Section 3. The conclusion is given in Section 4.

## 2. Proposed Ship Detection Method

Considering the real-time and accuracy requirements for practical detection applications, we implement the ship detection in two stages. In the first stage, the ship candidate regions are generated quickly by the prescreening algorithms. In the second stage, the correctness of each candidate region is verified by the proposed LCVWIE method.

As the ships are mostly made of metal materials and they usually have large hulls containing a lot of dihedral and trihedral structures, ships usually present as bright regions in SAR images. As a result, the ship region can keep stable at a certain gray level range, which coincides with the features of the MSER region. Therefore, the candidate region can be defined as the MSER region, and the prescreening method in the first stage can adopt the MSER method.

Since strong clutter can form bright regions in a SAR image, the candidate regions obtained by the MSER method may contain false alarms. It is necessary to verify these candidate region. Generally, ship target regions have distinct gray values in a SAR image. Moreover, the target regions have strong dissimilarity with respect to their surrounding regions, while the strong clutter regions usually have similarities with their surrounding regions. Considering the features of the candidate region, variance weighted information entropy (VWIE) [26] is an effective method to describe the difference of gray level and the influence of high gray levels in the candidate region. Meanwhile, considering the dissimilarity relationships between the candidate region and its surrounding regions, the local contrast measure (LCM) [22] is introduced to verify the candidate region along with VWIE. Consequently, a new LCVWIE method is proposed in the second stage for validating candidate regions.

From the above, we first prescreen the input image with the MSER method and obtain the candidate regions. Then, the proposed LCVWIE method is used to verify the candidate regions, and the final detection result is obtained by a threshold decision. The method is specified in the following section.

### 2.1. Candidate Region Generation

The MSER method is generally used to calculate image-stable regions based on the idea of the watershed in geography. The stable regions are solely defined by an extremal property of the intensity function in the region and on its outer boundary [23]. The MSER method is explained specifically as follows.

**Figure 1:**
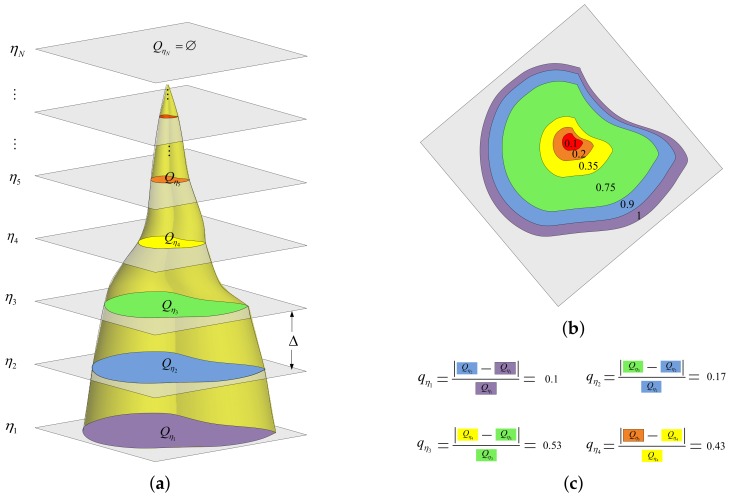
Calculation of the maximally-stable extremal region (MSER) region: (**a**) extremal regions’ generation; (**b**) top view of extremal regions; and (**c**) area variation rate calculation for extremal regions.

For a given intensity image, assume that its gray level range is [0,I]; *N* equal-interval thresholds of the gray level are set as $\left\{\eta_{i}|\eta_{i+1}=\eta_{i}+\Delta ,\eta_{i}\in \left[0,I\right],i=1,2,\ldots ,N\right\}$, where Δ means the interval and $\eta_{i}$ is the *i*-th threshold. For a certain threshold, if the gray level of the pixel in the image is smaller than the threshold, then the gray level of the pixel is set to zero; otherwise, it is set as one. In this way, we can get *N* binary images corresponding to the thresholds, and the region with a higher gray level in each binary image is called the extreme region. Specifically, the extreme region is a connected area corresponding to a certain gray level threshold, and the gray levels of all pixels in this area are larger than the threshold, while the gray levels outside this area are all smaller than the threshold. Then, a series of mutually extreme regions are generated, which are represented as $Q_{\eta_{1}}⊃Q_{\eta_{2}}⊃Q_{\eta_{3}}⊃\ldots ,⊃Q_{\eta_{N-1}}⊃Q_{\eta_{N}}$ in Figure 1.

An extremal region is stable if it satisfies the requirements shown in Equation (1):

(1) $$\left\{\begin{array}{c} S\left(Q_{\eta_{i}}\right)\in \left[a,b\right]\\ q_{\eta_{i}}=\frac{\left|S\left(Q_{\eta_{i+1}}\right)-S\left(Q_{\eta_{i}}\right)\right|}{S\left(Q_{\eta_{i}}\right)}<\varepsilon \end{array}\right.$$

where $S\left(\cdot \right)$ represents the area of the region, $\left[a,b\right]$ is the range of the area size, $q_{\eta_{i}}$ means the area variation rate of extremal region $Q_{\eta_{i}}$ and ε is the upper limit of the area variation rate.

In practice, it is very important to select appropriate parameters. The interval $\left[a,b\right]$ is estimated by the area range of ships and SAR image spatial resolution. This means that the range of the area size parameter should cover the area sizes of the vast majority of ships navigating in monitoring the ocean. As for the setting of Δ, a large Δ is too coarse to reflect the accuracy of $q_{\eta_{i}}$, while the small Δ will increase the calculation burden. Considering the tradeoff between the calculation efficiency and accuracy of $q_{\eta_{i}}$, Δ can be set between 10 and 16. For the intensity image of the ship target, the larger the Δ is, the bigger the ship’s area variation rate is. When Δ is fixed, too large a ε will screen out a large number of candidate regions, which is disadvantageous for calculation efficiency. Conversely, too small a ε will leave out dominated area variation, which is detrimental to target detection. Practically, the range of ε can be set as 0.2 to 0.4, which have been tested to be suitable for most of the SAR images.

It is obvious that the smaller the area variation rate is, the more stable the region is. Therefore, the maximally-stable extremal region is defined as the extremal region that minimizes the area variation rate that satisfies Equation (1). Using mathematical expression, that is:

(2) $$Q_{\mathrm{MSER}}=\underset{Q_{\eta_{i}}}{\arg\min}\left\{q_{\eta_{i}},i=1,2,\ldots \right\}$$

Figure 1 shows the calculation of the MSER region, where Figure 1a represents the generation of extremal regions, Figure 1b is the top view of Figure 1a and gives the normalized area of the mutually extreme regions and Figure 1c gives an example of the calculation to determine the MSER region. Take Figure 1 as an example: assume the normalized area range of a ship is set as [0.25,1], and ε is set to 0.45. According to Equation (1), the extremal regions $Q_{\eta_{5}}$ to $Q_{\eta_{N}}$ do not satisfy the area constraint. In the remaining extremal regions, the $Q_{\eta_{3}}$’s area variation rate $q_{\eta_{3}}>\varepsilon$, so it does not satisfy the area variation rate constraint. In the remaining extremal region $\left\{Q_{\eta_{1}},Q_{\eta_{2}},Q_{\eta_{4}}\right\}$, as $q_{\eta_{1}}<q_{\eta_{2}}<q_{\eta_{4}}$, $Q_{\eta_{1}}$ is selected as the MSER region, according to Equation (2), which is also the final target candidate region.

By the MSER method, the potential target regions can be obtained by prescreening the input SAR image. As the MSER method only require a small amount of computational resource, the computational efficiency of ship detection can be significantly improved. However, in high a sea state, rough sea surfaces are stirred by the fierce wind, which will lead to strong scattering returns for SAR. These strong scatter regions caused by sea waves are similar to the ship regions in the SAR image, which easily cause false alarms. In order to reduce the false alarms, the candidate regions should be verified, which is described in the following section.

### 2.2. Candidate Region Verification

In this section, the LCVWIE method is proposed to measure the candidate regions obtained in Section 2.1 by comprehensively considering the features of the candidate region and the relationship between the candidate region and its surrounding regions. Then, the final ship detection result is implemented by comparing the LCVWIE values of the candidate regions with the proposed adaptive threshold. Finally, the whole flowchart of the proposed ship detection method is given.

#### 2.2.1. LCVWIE Method

Assuming *K* candidate regions obtained by the MSER method are denoted as $u_{1},u_{2}\cdots u_{K}$, whose gray level ranges from zero to *I*, the VWIE of the *k*-th candidate region $u_{k}$ can be expressed as:

(3) $$H_{VWIE}^{\left(k\right)}=-\sum_{i=0}^{I}{\left(i-{\bar{I}}^{\left(k\right)}\right)}^{2}P_{k}\left(i\right)\log_{2}P_{k}\left(i\right)$$

where ${\bar{I}}^{\left(k\right)}$ denotes the mean gray level of the *k*-th candidate region, $P_{k}\left(i\right)$ means the occurrence probability of the pixels with gray level *i* in the *k*-th candidate region and $\sum_{i=0}^{I}P_{k}\left(i\right)=1$. It is stipulated that if $P_{k}\left(i\right)$ is equal to zero, then $P_{k}\left(i\right)\log_{2}P_{k}\left(i\right)$ is set to zero. In terms of ship detection from the SAR image, the smaller the $H_{VWIE}^{\left(k\right)}$ is, the more likely that the *k*-th candidate region contains the ship target.

**Figure 2:**
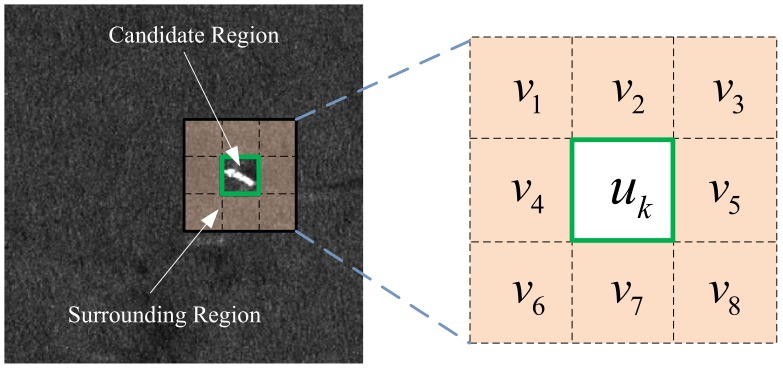
Illustration of the local contrast calculation stencil.

To take the local contrast characteristics of the candidate region into account, the surrounding regions for each candidate region should be constructed. Figure 2 shows the illustration of the local contrast calculation stencil. As shown in Figure 2, $u_{k}$ denotes the *k*-th candidate region obtained by the MSER method, and $\left\{v_{1},v_{2},\ldots ,v_{8}\right\}$ represents the surrounding regions corresponding to $u_{k}$. Assuming the mean gray level of the *j*-th surrounding region $v_{j}$ is $m_{v_{j}}$, it can be expressed as follows:

(4) $$m_{v_{j}}=\frac{1}{N_{v_{j}}}\sum_{n=1}^{N_{v_{j}}}I_{n}^{v_{j}}$$

where $N_{v_{j}}$ denotes the total number of the pixels in region $v_{j}$, and $I_{n}^{v_{j}}$ is the gray level of the *n*-th pixel in region $v_{j}$. The contrast between the central region $u_{k}$ and the *j*-th surrounding region is calculated by Equation (5).

(5) $$c_{v_{j}}^{k}=\frac{U_{k}}{m_{v_{j}}}$$

where $U_{k}$ represents the maximum gray level in central region $u_{k}$, $U_{k}=\max_{n=1,2,\ldots ,N_{u_{k}}}\left\{I_{n}^{u_{k}}\right\}$, $N_{u_{k}}$ means the total number of the pixels in region $u_{k}$ and $I_{n}^{u_{k}}$ denotes the gray level of the *n*-th pixel in region $u_{k}$. Then, the LCM value of the *k*-th candidate region $u_{k}$ is defined as:

(6) $$\begin{array}{c} C_{k}=\min_{v_{j}}U_{k}\times c_{v_{j}}^{k}\\ \;\;\;\;\;\;=\min_{v_{j}}U_{k}\times \frac{U_{k}}{m_{v_{j}}}\\ \;\;\;\;\;\;=\min_{v_{j}}\frac{U_{k}^{2}}{m_{v_{j}}} \end{array}$$

As the LCM value reflects the contrast between the central region and its surrounding regions, the bigger the $C_{k}$ is, the more dissimilar the central region to its neighborhoods is, which means the central region is more likely to be a ship region. Once the LCM values of all the *K* candidate regions have been calculated, the normalized LCM of the *k*-th candidate region can be given by Equation (7). The normalized LCM ranges from zero to one.

(7) $${\bar{C}}_{k}=\frac{C_{k}}{\max_{h=1,2,\ldots ,K}\left\{C_{h}\right\}}$$

Then, the VWIE value of the *k*-th candidate region can be modified by the normalized LCM, which is called LCVWIE and can be written as:

(8) $$H_{LCVWIE}^{\left(k\right)}={\bar{C}}_{k}\cdot H_{VWIE}^{\left(k\right)}$$

From Equation (8), it can be seen that the VWIE value of the central region is weighted by the normalized LCM value. In this way, the LCVWIE can not only describe the distribution of the gray level in the candidate region, but also reflects the dissimilarity between the candidate region and its surrounding regions. As the normalized LCM value of the false candidate regions caused by strong clutter is usually very small, the VWIE value of the false candidate region is reduced by LCM considerably, while the LCVWIE value of the ship region can still have a relatively large value. Then, through a threshold decision of LCVWIE, the false alarms and ship targets can be distinguished. This is how the LCVWIE method reduces false alarms of ship detection in the SAR image.

#### 2.2.2. Adaptive Threshold Calculation

In order to ensure the robustness of the proposed method, we adopt an adaptive LCVWIE threshold calculated from the whole SAR image, rather than a fixed one. Taking the characteristics of the SAR image into account, the LCVWIE threshold *T* is calculated by Equation (9).

(9) $$T=c\times H_{\omega }$$

where $H_{\omega }$ represents the VWIE value of the original SAR image and *c* is the adaptive adjustment parameter, which is calculated by $H_{\omega }$ as described in the literature [18]. As $H_{\omega }$ and *c* can adjust adaptively with the input SAR image, the threshold *T* determined by $H_{\omega }$ and *c* is adaptive. For the *k*-th candidate region, if $H_{LCVWIE}^{\left(k\right)}\geq T$, then the *k*-th candidate region is considered as a ship target, otherwise it is determined as a false alarm.

**Figure 3:**
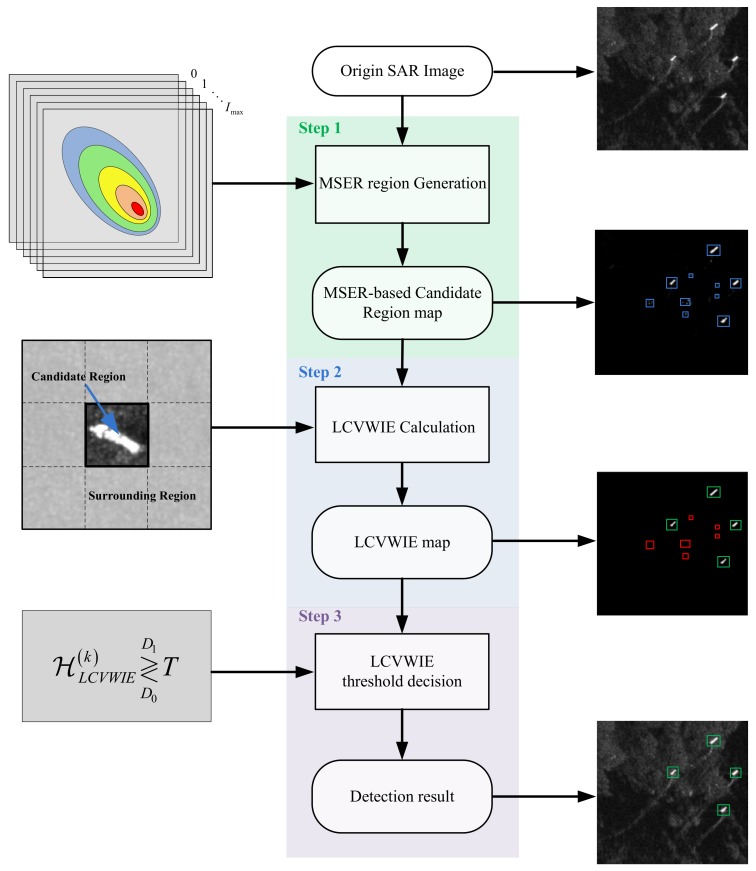
The flowchart of the proposed detection method. LCVWIE, local contrast variance weighted information entropy.

So far, the ship detection method has been described. The whole process of the proposed method is summarized in Figure 3.

Now, we analyze the computational complexity of the proposed method. In the candidate region generation stage, the MSER method adopts the BINSORTalgorithm [27] to implement pixel sorting, and after sorting, the efficient union-find algorithm [27] is used to maintain the list and area of connected regions [23]. The computational complexity of the BINSORT algorithm and the Union-Find algorithm are $O\left(N\right)$ and $O\left(N\log\log N\right)$, respectively; where *N* is the number of pixels in the original SAR image. Therefore, the candidate region generation step has an approximately linear complexity.

In the candidate region verification stage, the computational complexity is mainly determined by the calculation of the LCVWIE value in Equation (8). We assume that the image gray level is $\left[0,I\right]$, and a total of *K* candidate regions has been obtained by the MSER method, during which the largest candidate region contains $N_{t}$ pixels. For the convenience of illustration, it is reasonable to assume the time of performing one multiplication and that of performing one addition are equal. The complexity of the LCVWIE method is determined by the calculation complexities of Equations (3), (7) and (8). After some simple calculations, it can be obtained that the complexity of calculating $\left\{H_{VWIE}^{\left(k\right)},k=1,2,\ldots K\right\}$ in Equation (3) is $K\left(5I+2N_{t}\right)$, while $K\left(9N_{t}+15\right)+\left(2K-1\right)$ operations are needed to calculate $\left\{{\bar{C}}_{k},k=1,2,\ldots K\right\}$ in Equation (7). In addition, *K* operations have been done to obtain the LCVWIE value by multiplying Equations (3) and (7) in Equation (8). Therefore, the computational complexity of the LCVWIE method can be calculated as $K\left[11N_{t}+5I+18\right]-1$ operations. In general, the density of the vessels in the sea scene is very low [28], which means $KN_{t}\ll N$. Therefore, the total computational complexity of the algorithm is close to $O\left(N\right)$.

Based on the analysis above, it can be concluded that the proposed method has approximately linear complexity, which is very important for real-time processing.

## 3. Experiments and Results

**Figure 4:**
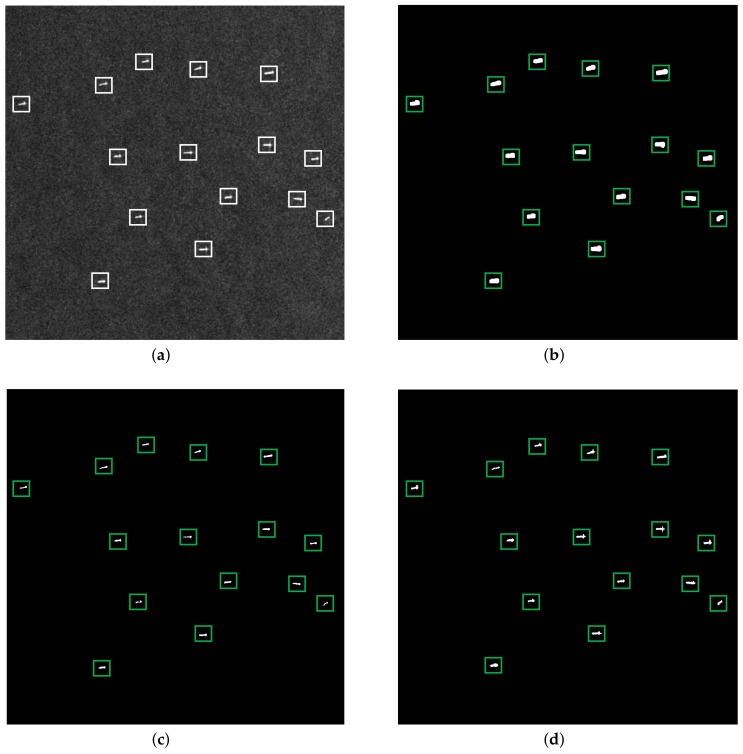
Detection results of a homogeneous scene: (**a**) original SAR image, (**b**) detection result of the VWIE method, (**c**) detection result of the CA-CFAR method and (**d**) detection result of the proposed method.

**Figure 5:**
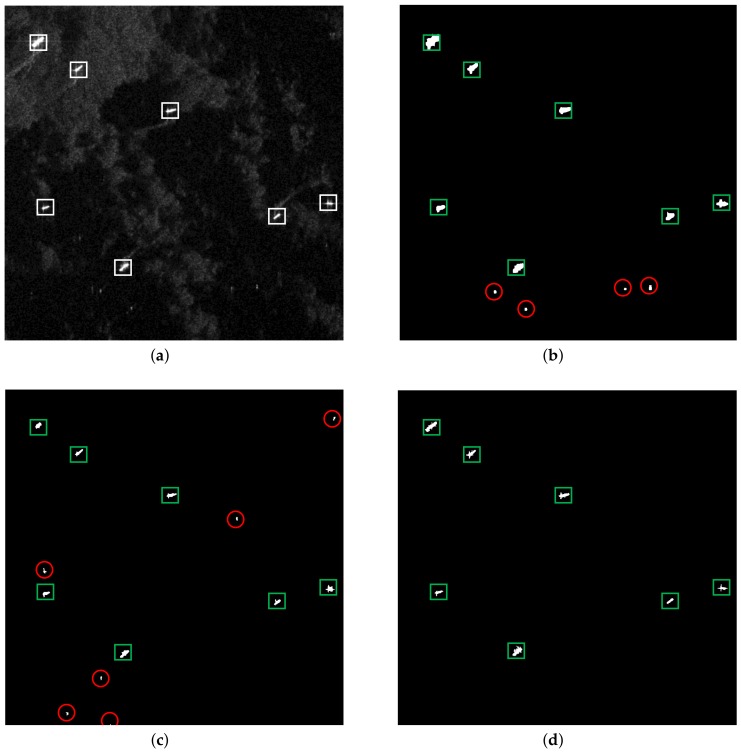
Detection results of a heterogeneous scene: (**a**) original SAR image, (**b**) detection result of the VWIE method, (**c**) detection result of the CA-CFAR method and (**d**) detection result of the proposed method.

**Figure 6:**
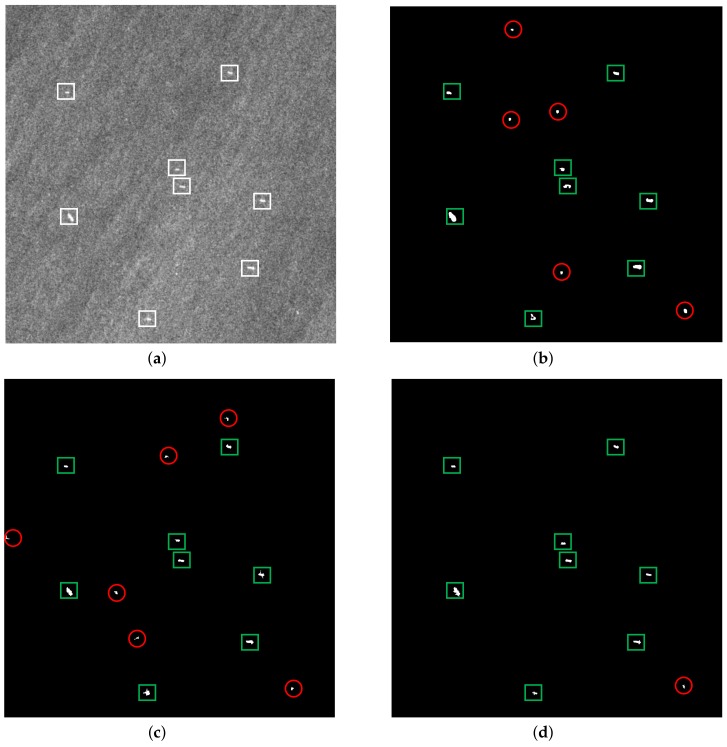
Detection results of the strong clutter scene: (**a**) original SAR image, (**b**) detection result of the VWIE method, (**c**) detection result of the CA-CFAR method and (**d**) detection result of the proposed method.

In order to verify the proposed detection method, we execute experiments with three ocean SAR images containing different scenes, namely a homogeneous scene, a heterogeneous scene and a strong clutter scene, which are shown in Figure 4a, Figure 5a and Figure 6a, respectively. The SAR images of homogeneous and heterogeneous scenes come from Sentinel-1A C-band SAR images with an image size of 1000×1000 pixels and a 10-m pixel size. The SAR image of the strong clutter scene comes from ERS C-band SAR image with an image size of 1000×1000 pixels and a 12.5-m pixel size.

In the experiments, the proposed method will be compared with the VWIE method [18] and CA-CFAR method based on the $G^{0}$ distribution [29], and the detection performance and computational efficiency of these methods are analyzed. The area range of ship target is set as [3,300], and the area variance rate threshold is set to 0.3 for the proposed method. In the VWIE method, the window size ranges from six to nine. The CA-CFAR method is presented at a false alarm probability of $10^{-4}$, and the sliding window size is 71, where the clutter ring window size is 15 and the guard ring window size is 20.

### 3.1. Experiment Results

#### 3.1.1. Homogeneous Scene

Figure 4 shows the detection results of the three methods under a homogeneous background. The original SAR image of the homogeneous scene is shown in Figure 4a, which contains 15 ships, labeled by white rectangles. Figure 4b–d demonstrate the detection results of the VWIE method, the CA-CFAR method and the proposed method, respectively, where the green rectangle means the correct detection. As the signal-to-clutter ratio (SCR) is high for a homogeneous scene, the three methods successfully detected the ship targets without false alarm and missing inspection.

It should be noted that as the sliding window containing part of the target will lead to a large VWIE value, the VWIE method may cause the phenomenon of target boundary broadening [18]. By contrast, the proposed method shows promising detection results.

#### 3.1.2. Heterogeneous Scene

In general, an ocean ship is made of metal, and its scattering could last a longer time than sea clutter in the azimuth [30], so its scattering intensity is stronger than the sea surface’s. As a result, the ship is brighter than the sea background in the SAR image, which is helpful for detecting the ship target. However, in a heterogeneous scene, the sea spikes may be as bright as ship targets in the SAR image. It is quite difficult to distinguish the ship target in this situation. Therefore, examining the performance of the detectors under heterogeneous background conditions is quite necessary.

Figure 5 shows the detection results of three methods under a heterogeneous background. Figure 5a is the original SAR image of the heterogeneous scene, which contains seven ships. Figure 5b–d present the detection results of the three detection methods, respectively, where the green rectangle means the correct detection and the red circle represents the false alarm. It can be seen that the VWIE and the CA-CFAR methods lead to false alarms to different degrees. In contrast, the proposed method shows satisfactory detection performance, which means the proposed method has sufficient applicability under a heterogeneous background.

#### 3.1.3. Strong Clutter Scene

A strong clutter background is a common phenomenon when SAR observes the sea surface at a high grazing angle, and the SCRwill become lower in this scenario, which is a big challenge for ship detection. Consequently, the performance of the detector in this scenario should be investigated. Figure 6 shows the detection results of the three methods under a strong clutter background. The original SAR image of the strong clutter scene is shown in Figure 6a, which contains eight ships.

Figure 6b–d give the detection results of the three detection methods, respectively, where the green rectangle means the correct detection and the red circle represents the false alarm. It can be seen in Figure 6 that the proposed method has less false alarms than the VWIE method and the CA-CFAR method. In contrast to the other two methods, the proposed method takes the local contrast between the target regions and the surrounding regions into account, which can effectively distinguish the target and the clutter, and achieves better detection results.

**Figure 7:**
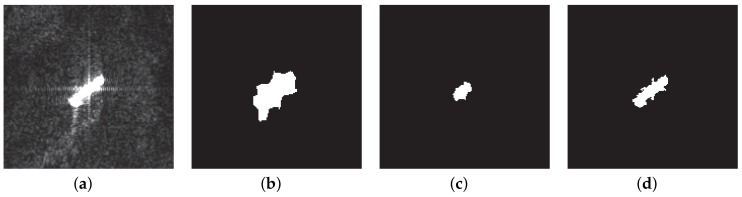
Detection results of one ship slice: (**a**) original ship slice, (**b**) detection result of the VWIE method, (**c**) detection result of the CA-CFAR method and (**d**) detection result of the proposed method.

Figure 7 shows the detection results of one ship slice in the experiments. The original SAR image of the ship slice is shown in Figure 7a–d, giving corresponding detection results of the three detection methods, respectively. It can be seen from the results that the VWIE method makes the ship boundary broaden, and a shrinkage of the ship area has appeared in the $G^{0}$-based CA-CFAR method. In contrast, the proposed method basically maintained the original boundary and area of the ship.

### 3.2. Performance Analysis

#### 3.2.1. Detection Performance

To evaluate the detection performance of the proposed method and the other two methods, the figure-of-merit (FoM) [31] is introduced to assess the detection results. The FoM of the detection result can be calculated by Equation (10).

(10) FoM=NcdNcdNfa+NttNfa+Ntt

where $N_{cd}$ means the number of correct detections, $N_{fa}$ denotes the number of false alarms and $N_{tt}$ is the number of real targets in the original SAR image. It is obvious that the bigger the FoM is, the more accurate the ship detection results are.

Table 1 shows the FoMs of detection results for the three methods. From Table 1, it can be seen that the proposed method performs much better than the VWIE method and CA-CFAR method in the three sea scenes. Moreover, The variances of the FoM for the VWIE method, the CA-CFAR method and the proposed method are 0.0312, 0.0443 and 0.0027, respectively. It is clear that the FoM variance of the proposed method is significantly smaller than the other two methods, which means that the proposed method has better robustness.

#### 3.2.2. Computational Efficiency

Computational efficiency is a key indicator of the real-time processing performance for a detection algorithm. In order to evaluate the computational efficiency of the three detection methods, all the experiments are accomplished by MATLAB R2015b on a computer with the hardware environment of Pentium Dual-Core CPU i3-3220 3.3 GHz and 8 GB RAM. Therefore, the computational time of each method can measure the computational efficiency. Table 2 shows the computational times of the three methods. The computational times of the proposed method are sharply reduced compared with the other two methods, which makes the proposed method available in the application of real-time ship detection.

From the experimental results and performance analysis, it can be seen that our method has the advantages of accurate detection, strong robustness and high computational efficiency, which proves that it is valuable in practical application.

## 4. Conclusions

In this paper, an effective ship detection method is proposed to detect the ship targets from SAR images. Considering the real-time and accuracy requirements for the practical detection applications, we implement the ship detection in two stages. First, the ship candidate regions are generated by the MSER method. Then, the LCVWIE method is proposed to validate the candidate regions by taking the characteristics of the ship regions into account. Compared with the conventional methods, the proposed method does not require any prior knowledge, and it is available for heterogeneous and strong clutter scenes. Moreover, the computational consumption is sharply reduced. Experiments are carried out based on the spaceborne SAR images, and the results demonstrate that the proposed method can achieve more accurate detection results with much less computational time in three different sea scenes than some existing detection methods. The ship detection performance of the proposed method makes it able to be applied to the field of marine surveillance.

However, there is still room for improvement. As the proposed method adopts image processing technology, too small targets may be missed in the processing of the morphology. Additionally, in harsh weather conditions, ships may present a backscattering value similar to the surrounding sea clutter, leading to degradation of detection performance. In the future, we will research the detection for small targets, explore an approach to realize effective ship detections in harsh weather conditions and verify the influence of azimuth ambiguities. Furthermore, polarization information can be introduced to improve the detection performance [32,33].

## Figures and Tables

**Table 1:** FoMs of the detection results for the three methods.

	$N_{\mathrm{cd}}$	$N_{\mathrm{fa}}$	$N_{\mathrm{tt}}$	FoM
Homogeneous scene (Figure 4)
VWIE method	15	0	15	1
CA-CFAR method	15	0	15	1
Proposed method	15	0	15	1
Heterogeneous scene (Figure 5)
VWIE method	7	4	7	0.636
CA-CFAR method	7	6	7	0.538
Proposed method	7	0	7	1
Strong clutter scene (Figure 6)
VWIE method	8	5	8	0.615
CA-CFAR method	8	6	8	0.571
Proposed method	8	1	8	0.889

**Table 2:** Computational times of the three detection methods.

	Proposed Method	VWIE Method	CA-CFAR Method
Homogeneous scene	1.55 s	143.06 s	167.16 s
Heterogeneous scene	1.52 s	144.31 s	159.61 s
Strong clutter scene	1.36 s	148.09 s	142.01 s
